# DLX6-AS1: A Long Non-coding RNA With Oncogenic Features

**DOI:** 10.3389/fcell.2022.746443

**Published:** 2022-02-25

**Authors:** Soudeh Ghafouri-Fard, Sajad Najafi, Bashdar Mahmud Hussen, Aryan R. Ganjo, Mohammad Taheri, Mohammad Samadian

**Affiliations:** ^1^ Department of Medical Genetics, School of Medicine, Shahid Beheshti University of Medical Sciences, Tehran, Iran; ^2^ Department of Medical Biotechnology, School of Advanced Technologies in Medicine, Shahid Beheshti University of Medical Sciences, Tehran, Iran; ^3^ Department of Pharmacognosy, College of Pharmacy, Hawler Medical University, Kurdistan Region, Erbil, Iraq; ^4^ Center of Research and Strategic Studies, Lebanese French University, Erbil, Iraq; ^5^ Urology and Nephrology Research Center, Shahid Beheshti University of Medical Sciences, Tehran, Iran; ^6^ Institute of Human Genetics, Jena University Hospital, Jena, Germany; ^7^ Skull Base Research Center, Loghman Hakim Hospital, Shahid Beheshti University of Medical Sciences, Tehran, Iran

**Keywords:** DLX6-AS1, non-coding RNA, lncRNA, cancer, miRNA 3

## Abstract

Long non-coding RNAs (lncRNAs) are a heterogeneous group of ncRNAs with characteristic size of more than 200 nucleotides. An increasing number of lncRNAs have been found to be dysregulated in many human diseases particularly cancer. However, their role in carcinogenesis is not precisely understood. DLX6-AS1 is an lncRNAs which has been unveiled to be up-regulated in various number of cancers. In different cell studies, DLX6-AS1 has shown oncogenic role *via* promoting oncogenic phenotype of cancer cell lines. Increase in tumor cell proliferation, migration, invasion, and EMT while suppressing apoptosis in cancer cells are the effects of DLX6-AS1 in development and progression of cancer. In the majority of cell experiment, mediator miRNAs have been identified which are sponged and negatively regulated by DLX6-AS1, and they in turn regulate expression of a number of transcription factors, eventually affecting signaling pathways involved in carcinogenesis. These pathways form axes through which DLX6-AS1 promotes carcinogenicity of cancer cells. Xenograft animal studies, also have confirmed enhancing effect of DLX6-AS1 on tumor growth and metastasis. Clinical evaluations in cancerous patients have also shown increased expression of DLX6-AS1 in tumor tissues compared to healthy tissues. High DLX6-AS1 expression has shown positive association with advanced clinicopathological features in cancerous patients. Survival analyses have demonstrated correlation between high DLX6-AS1 expression and shorter survival. In cox regression analysis, DLX6-AS1 has been found as an independent prognostic factor for patients with various types of cancer.

## Introduction

In complex organisms, genome sequencing analyses have unveiled that just a small fraction of genome (e.g., 1–2% for mammals) encodes for protein *via* coding RNAs or messenger RNAs (mRNAs) that are located in the middle of central dogma making connection between DNA and corresponding protein. These protein-coding regions are those which have been described as genes for more than half a century in biology literature. However, the majority of large genomes i.e., more than 80% is transcribed to non-coding RNAs (ncRNAs) for which no corresponding protein have been found, but a huge number of regulatory functions are recognized. Unlike the primary expectations which termed ncRNAs as “junk” DNA without biological importance, today it is clarified that they are involved in gene regulation at transcriptional and post-transcriptional levels, and through which they play critical roles in a vast number of biological processes such as imprinting, methylation, and silencing *via* several interactions with DNA, RNA, and proteins (Mattick, 2001). Based on size and function of transcripts, ncRNAs are categorized in several classes including microRNAs (miRNAs), small interfering RNAs (siRNAs), PIWI-interacting RNAs and long ncRNAs (lncRNAs). Transcripts of more than 200 nucleotide length are classified as lncRNAs which were primarily reported by Okazaki *et al.* in an analysis of mouse transcriptome in 2002 (Okazaki et al., 2002). RNA polymerase II is predominantly responsible for transcription of lncRNAs. They mainly endure capping, polyadenylation, splicing after transcription, and also trimethylation on histone 3 corresponding to lysine 4 (H3K4me3) (Losko et al., 2016; Bertone et al., 2004; Guttman et al., 2009). Thousands of heterogenous lncRNAs have been identified in multicellular organisms [60,000 encoding loci in human genome (Iyer et al., 2015)] showing tissues specificity which is also conserved during evolution (Necsulea et al., 2014) and acting as regulators of gene expression both in nucleus or cytoplasm (Fatica and Bozzoni, 2014) suggesting their involvement in specific biologic processes. Several databases have been created to store and provide access to an increasing number of lncRNAs. Examples of these databases are TRlnc for regulatory lncRNAs in humans (Li et al., 2020a), lncRNASNP1 and 2 for single nucleotide polymorphisms (SNPs) of human and mouse lncRNAs (Gong et al., 2014; Miao et al., 2017), LncRNA2Target v2.0 for target genes of lncRNAs (Cheng et al., 2018), CRISPRlnc for validated single guide RNAs (sgRNAs) used in clustered regularly interspaced short palindromic repeats (CRISPR)-associated protein number 9 (Cas9) gene editing technology for lncRNAs (Chen et al., 2018) and clusLnc2Cancer for effective lncRNAs in human cancers (Ning et al., 2015). They act in *cis* and *trans* modes by gathering and localizing transcription factors to a locus. Gene expression regulation at several levels including transcription, translation and splicing, epigenetic regulation in X-chromosome inactivation or dosage compensation, genomic imprinting, involvement in developmental and differential processes, neurogenesis, regulation of cell cycle, and cell transportation are among the fundamental roles which have been recognized for lncRNAs (Mattick, 2009; Wilusz et al., 2009; Wu et al., 2013; Dey et al., 2014; Fatica and Bozzoni, 2014). Accordingly, an increasing number of lncRNAs have been associated with various types of human diseases. Dysregulation in expression levels or mutation of lncRNAs are found to play role in the pathogenesis of diseases like age-related diseases, cardiovascular diseases (Uchida and Dimmeler, 2015), kidney and liver diseases (Takahashi et al., 2014; Ignarski et al., 2019), ophthalmologic diseases (Wawrzyniak et al., 2018), neurodegenerative and other diseases affecting central nervous system (CNS) (Pastori and Wahlestedt, 2012; Wan et al., 2017), and particularly various types of cancer. Mediation of a number of cancer-associated processes like cell cycle regulation, epigenetic regulation, and involvement in signaling pathways and hormone-related pathways indicate potential roles of lncRNAs act as contributors in the development and progression of cancer (Sahu et al., 2015). MALAT1, HOTAIR, H19, HOTTIP, ANRIL, and NEAT1 are among the most famous lncRNAs which have been mostly studied in many types of cancer exhibiting dysregulation in cancer cells, tissues and body fluids of affected patients. In this review, we aim to have an overview of studies which have assessed tumorigenic effects of the lncRNA distal-less homeobox 6 antisense RNA 1 (DLX6-AS1) in three levels of cell, animal, and human studies. In humans, *DLX6-AS1* gene is located on chromosome 7q21.3, primarily identified by Feng et al. (2006) to promote DLX5/6 function in *trans* mode. This lncRNA has been found to be up-regulated in a growing number of different types of cancerous tissues compared to normal tissues. Promoting carcinogenesis *via* increasing tumor cell proliferation, migration, and invasion through enhancing Epithelial–Mesenchymal Transition (EMT) along with suppression of apoptosis and chemosensitivity have been shown in cell studies of DLX6-AS1 overexpression. Enhanced tumor growth and metastasis has confirmed tumorigenic potentials of DLX6-AS1 in animal studies. Correlation between high DLX6-AS1 expression and advanced clinicopathological features and also poor prognosis and survival in cancerous patients has suggested DLX6-AS1 not only as a diagnostic and prognostic biomarker but also as a therapeutical target.

### Functional Effects of DLX6-AS1 on Cell Proliferation, Apoptosis and Migration

Cancer cell lines have been used to evaluate function of DLX6-AS1 in cell cycle progression, cell proliferation and apoptosis. Moreover, high throughput RNA sequencing and also confirmation *via* quantitative real-time polymerase chain reaction (qRT-PCR) analyses have facilitated identification of differentially expressed lncRNAs in cancer cell lines compared to controls. *In vitro* experiments have shown significant increase in expression levels of DLX6-AS1 in cancer cell lines. In different cell experiment, it has been demonstrated that DLX6-AS1 overexpression promotes tumor cell proliferation, migration, and invasion, while suppressing apoptosis. In cell counting, colony formation, and 5-Bromo-2-deoxyUridine (BrdU) assays, decreased proliferation of cancer cells is reported for DLX6-AS1 knockdown. Wound healing, Matrigel and Transwell assays for assessment tumor cell migration and invasion show suppressed metastatic capability of cancerous cells under DLX6-AS1 silencing. Flowcytometry also demonstrated cell cycle arrest in treated cancer cells. Furthermore, decreased cell viability and elevated apoptosis in 3-(4,5-dimethylthiazol-2-yl)-2,5-diphenyl-2H-tetrazolium bromide (MTT), flowcytometry, and apoptotic marker assays have unveiled increased apoptosis in DLX6-AS1-silenced cancer cells. In hepatocellular carcinoma (HCC), DLX6-AS1 has been shown to be highly expressed in human HCC cell lines versus normal liver cells, while miR-513c as its downstream microRNA exhibited down-regulation indicating DLX6-AS1 acts as sponge for this miRNA (Liu et al., 2020a). Cullin4A (*Cul4A*) was also known as target gene of miR-513c which showed increase in expression level following DLX6-AS1 up-regulation. In other words, DLX6-AS1 elevated *Cul4A* expression by binding to and sponging miR-513c. Cul4A, itself positively regulated activity of annexin A10 (*ANXA10*). DLX6-AS1 silencing using specific short hairpin RNA (shRNA) repressed cell viability, invasion, and migration of HCC cells. Also, Cul4A knockdown was shown to inhibit tumorigenic effects of HCC cells *via* inhibition of ANXA10 degradation through ubiquitin-associated pathway. The results showed that DLX6-AS1 exerts its tumorigenic role *via* miR-513c/Cul4A/ANXA10 axis. In a distinct study (Zhang et al., 2017), DLX6-AS1 was shown to exert same tumorigenic roles in HCC cells *via* miR-203a/MMP-2 axis.

In other experiments, DLX6-AS1 has been shown to sponge many other miRNAs and affect transcription factors, genes or signaling pathways which eventually promotes malignant phenotypes. miRNAs which are mainly negatively regulated by up-stream DLX6-AS1 exhibit down-regulation in cancer tissues and cells, and their overexpression reverse the malignant phenotypes of DLX6-AS1 in cancer cell lines. Downstream factors demonstrate expression changes consistent with DLX6-AS1. Overexpression of these factors drives same influences with DLX6-AS1 overexpression. In a study in ovarian cancer (Kong and Zhang, 2020), miR-195-5p was shown to be down-regulated in cancer tissues and was identified as target of up-regulated DLX6-AS1. While DLX6-AS1 promoted cell proliferation, migration, and invasion in tumor cell lines, miR-195-5p overexpression reversed malignant phenotypes. Four and a half LIM domains protein 2 (FHL2) which is known to play role in development and progression of different types of cancer *via* activation of androgen receptor (AR or NR3C4), Wnt/β-catenin pathway or several genes was demonstrated as target of miR-195-5p. FHL2 overexpression exhibited same results on malignant phenotypes of cancer cells. In other words, DLX6-AS1 exerted its tumorigenic effects in ovarian cancer cells *via* miR-195-5p/FHL2 signaling axis. In bladder cancer, miR-195-5p as target of DLX6-AS1 was shown to down-regulate the vascular endothelial growth factor A (VEGFA) and consequently inhibit malignancy phenotype in cancer cells, while miR-195-5p inhibition returned the DLX6-AS1 tumorigenic effects (Zhao et al., 2020a).

Furthermore, DLX6-AS1 has been shown to up-regulated DLK1, a regulator of cell differentiation and prognostic factor for several cancers, through sponging miR-129-5p which in turn triggers Wnt signaling, and eventually promotes stemness in osteosarcoma cell lines (Zhang et al., 2018). PI3K/AKT/mTOR signaling pathway is another critical tumorigenic pathway which is known to be activated by DLX6-AS1, promoting malignant phenotype of colorectal cancer cells (Zhang et al., 2019a).

**FIGURE 1:**
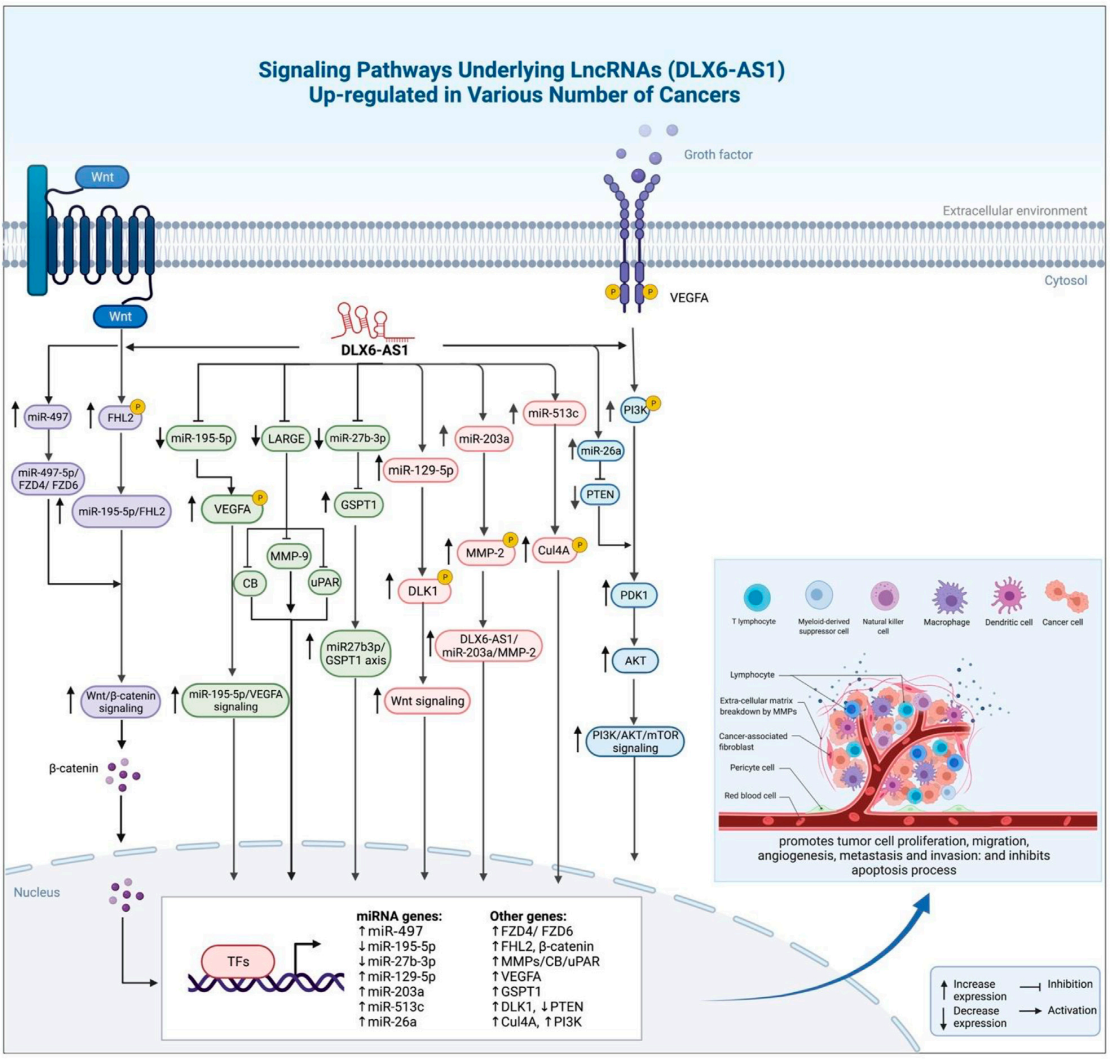
Oncogenic role of DLX6-AS1 in different cancer types is exerted through various mechanisms, particularly sponging miRNAs.

Overall, it is demonstrated that DLX6-AS1 acts as an oncogenic lncRNA enhancing malignant phenotype of several cancer cells (Figure 1).

DLX6-AS1 is oncogenic lncRNA has been found to be up-regulated in a growing number of different types of cancerous tissues compared to normal tissues. Promoting carcinogenesis *via* increasing tumor cell proliferation, migration, and invasion through enhancing Epithelial–Mesenchymal Transition (EMT). miRNAs have been identified which are negatively regulated by DLX6-AS1, and they regulate expression of a number of transcription factors, eventually affecting signaling pathways involved in carcinogenesis.

Table 1 shows the findings of the studies conducted on DLX6-AS1 oncogenic role in various cancer cell lines.

**TABLE 1:** an overview to the oncogenic influences of DLX6-AS1 in cell studies of different types of cancer.

Cancer type	Targets/Regulators and signaling pathways	Assessed cell lines	Function	References
HCC	miR-513c/Cul4A/ANXA10 axis	Hep3B, HepG2, Huh7, PLC/PRF/5, and THLE-3	Δ DLX6-AS1: ↓tumor cell viability, ↓invasion, and ↓migration	Liu et al. (2020a)
	miR-203a/MMP-2 axis	Hep3B, MHCC97L, HCCLM3, HepG2, Huh7, and LO2	Δ DLX6-AS1: ↓tumor cell proliferation, ↓invasion, and ↓migration	Zhang et al. (2017)
Pancreas	miR-181b/ZEB2 axis	CAPAN-1, BxPC-3, SW 1990, PANC-1, and HPDE6-C7	Δ DLX6-AS1: ↓tumor cell proliferation, ↓migration, and ↓invasion	An et al. (2018)
	miR-497-5p/FZD4/FZD6/Wnt/β-catenin axis	Panc-1, AsPC-1, Bxpc-3, Capan-1, CFPAC-1, and MIA PaCa-2	↑↑ DLX6-AS1: ↑tumor cell proliferation, ↑migration, and ↑invasion, while Δ DLX6-AS1 reversed the tumorigenic effects	Yang et al. (2019a)
Prostate	miR-497-5p/SNCG axis	LNCap, DU145, PC-3, VCap, and WPMY1	Δ DLX6-AS1: ↓tumor cell proliferation, ↑apoptosis	Zhu et al. (2021)
	DNMT1/LARGE axis	CWR22rv1, LAPC-9, DU145, LNCaP, PC-3M, and PrEC	↑↑ DLX6-AS1: ↑tumor cell proliferation, ↑migration, and ↑invasion	Zhao et al. (2020b)
Kidney (renal cell carcinoma; RCC)	miR-26a/PTEN axis	A498, ACHN, Caki-1, Caki-2, 786-O, G401, and HK-2	Δ DLX6-AS1: ↓tumor cell proliferation, and ↓colony formation	Zeng et al. (2017)
Liver	miR-424‐5p/WEE1 axis	MHCC97L, HCCLM3, SK‐HEP‐1, Hep3B, Huh7, and HEK293T	Δ DLX6-AS1: ↓tumor cell proliferation, ↓migration, and ↓invasion	Li et al. (2019a)
	CADM1/STAT3 axis	Hep3B, HepG2, SMMC-7721, HCCLM3, Huh7 and L02	Δ DLX6-AS1: ↓self-renewal, ↓amplification, and ↓proliferation in liver cancer stem cells	Wu et al. (2019)
Neuroblastoma	miR-513c-5p/PLK4 axis	SK-N-SH, SK-N-AS NB, and HUVEC	Δ DLX6-AS1: ↓tumor cell viability, ↓colony formation, ↓migration, ↓invasion, ↑apoptosis and ↑cell cycle arrest	Jia et al. (2020)
	miR-506-3p/*STAT2* axis	SK-N-SH and LAN-6	Δ DLX6-AS1: ↓tumor cell proliferation, ↓glycolysis and ↑ cell cycle arrest at G1/S phase	Han et al. (2020)
	miR-497-5p/YAP1 axis	SK-N-AS, SK-N-SH, SH-SY5Y, and SK-N-BE	Δ DLX6-AS1: ↓tumor cell proliferation, ↓migration, ↓invasion, and ↓EMT	Li et al. (2020b)
	miR-107/BDNF axis	NB-1643, SK-N-SH, NB-1691, SK-N-AS, IMR-32, and SH-SY5Y	Δ DLX6-AS1: ↓tumor cell proliferation, ↓migration, ↓invasion, and ↑apoptosis	Li et al. (2019b)
Glioma	miR-197-5p/E2F1 axis	U251, T98G, U87MG, SHG44, and NHA	Δ DLX6-AS1: ↓tumor cell proliferation, and ↓invasion	Zhang et al. (2019b)
Osteosarcoma	miR-129-5p/DLK1 axis	MG63 and U2OS	Δ DLX6-AS1: ↓ number and size of tumor spheres, and ↓CSCs in osteosarcoma cell lines	Zhang et al. (2018)
			DLX6-AS1 triggers Wnt signaling	
	miR-641/HOXA9 axis	Saos‐2, MG‐63, U2OS and hFOB	Δ DLX6-AS1: ↓tumor cell proliferation, ↓migration, ↓invasion, and ↑apoptosis	Zhang et al. (2019b)
Endometria	DLX6	HEC-1‐B, HHUA, HEC‐1‐A, RL‐952, and HEC‐251	Δ DLX6-AS1: ↓tumor cell proliferation, ↓invasion, and ↑apoptosis	Zhao and Xu, (2020)
			DLX6-AS1 up-regulated DLX6 through inducing its promotor *via* p300/E2F1	
Cervix	miR-16-5p/ARPP19 Axis	SiHa, HeLa, C-33A, CaSki, and End1/E6E7	Δ DLX6-AS1: ↓tumor cell proliferation, ↓migration, ↓EMT and ↑apoptosis	Xie et al. (2020)
	miR-199a	CaSki, ME-180, C-33A, SiHa, HeLa, and NC104	Δ DLX6-AS1: ↓tumor cell proliferation, ↓colony formation, ↓migration, and ↑apoptosis	Long et al. (2019)
Breast	miR-505-3p/RUNX2 axis	MDA-MB-231, MDA-MB-468, BT-474, MCF-7, T47D, and MCF-10A	Δ DLX6-AS1: ↓tumor cell proliferation, ↓migration, ↓invasion, and ↑apoptosis	Zhao et al. (2019)
Breast (triple-negative; TNBC)	miR-199b-5p/paxillin axis	CCD-1095Sk, MDA-MB-231, HCC 1806, HCC1599, and HS578 T	Δ DLX6-AS1: ↓tumor cell proliferation, ↓EMT, ↑apoptosis, and ↓chemoresistance to cisplatin	Du et al. (2020)
Ovaries	miR-195-5/FHL2 axis	SKOV3, A2780, IOSE80, and 293 T	Δ DLX6-AS1: ↓ tumor cell proliferation, ↓migration, ↓invasion, and ↑apoptosis	Kong and Zhang, (2020)
	Notch	IOSE80, HEY, SKOV3, and OVCAR-3	Δ DLX6-AS1: ↓ tumor cell proliferation, ↓migration, ↓invasion, and ↑apoptosis	Zhao and Liu, (2019)
Bladder	miR-195-5p/VEGFA	T24, RT4, 5637, J82, SW780, and SV-HUC-1	Δ DLX6-AS1: ↓ tumor cell proliferation, ↓migration, ↓invasion, and ↑apoptosis	Zhao et al. (2020a)
	Wnt/β-catenin	5637, J82, T24, and SV-HUC-1	↑↑DLX6-AS1: ↑ tumor cell proliferation, ↑migration, ↑invasion, and ↑EMT. Knockdown reversed the malignancy phenotype of cells	Guo et al. (2019)
	miR-223/HSP90B1 axis	T24, SW780, and SV-HUC-1	Δ DLX6-AS1: ↓ tumor cell proliferation, and ↓invasion	Fang et al. (2019)
Colorectal	miR-26a/EZH2 Axis	DLD-1, HCT-116, HT-29, SW480, SW620, and NCM460	Δ DLX6-AS1: ↓ tumor cell proliferation, ↓migration, ↓invasion, and ↑cell cycle arrest	Kong et al. (2020)
	PI3K/AKT/mTOR pathway	HCT116, HT-29, SW480, and NCM460	↑↑ DLX6-AS1: ↑ tumor cell proliferation, ↑migration, ↑invasion, and ↓apoptosis. Δ DLX6-AS1 returned the malignant phenotype of cancer cells	Zhang et al. (2019a)
Larynx	miR-26a/TRPC3 axis	HEp-2 and Tu-177	Δ DLX6-AS1: ↓ tumor cell proliferation *via* decrease in mitochondrial radical oxygen species	Liu et al. (2020b)
			DLX6-AS1 regulates metabolism of cancer cells	
	miR-376c	Hep2	Δ DLX6-AS1: ↓ tumor cell proliferation, ↓invasion, and ↑cell cycle arrest	Yang et al. (2019b)
Nasopharynx	miR‐199a‐5p/HIF‐1α	S18, S26, CNE‐1, CNE-2, HONE‐1, 5‐8F, and NP69	Δ DLX6-AS1: ↓ tumor cell proliferation, ↓migration, and ↓invasion	Yang et al. (2020)
	axis			
Esophagus	--	EC109, KYSE30, and Het-1A	Δ DLX6-AS1: ↓ tumor cell proliferation, ↓migration, ↓invasion, and ↓EMT	Zhang et al. (2019c)
Stomach	miR-4290/PDK1 axis	HGC-27, SGC7901, MGC803, MKN45, and GES-1	Δ DLX6-AS1: ↓ tumor cell proliferation, ↑apoptosis, and caused glucose metabolism impairment	Qian et al. (2021)
	FUS/MAP4K1 axis	AGS, HGC-27, SGC-7901, BGC-823, and GES-1	Δ DLX6-AS1: ↓ tumor cell proliferation, ↓migration, and ↓EMT	Wu et al. (2020)
	miR-204-5p/OCT1 axis	MGC-803, HGC-27, MKN-7, MKN-28, MKN-45, AGS, SGC-7901, and GES-1	Δ DLX6-AS1: ↓ tumor cell proliferation, ↓migration, ↓invasion, and ↓EMT	Liang et al. (2020)
	--	HGC27, BGC823, SGC7901, AGS, and GES-1	Δ DLX6-AS1: ↓ tumor cell proliferation, ↓colony formation, ↓migration, ↓invasion, ↓EMT, and ↓cell cycle progression	Fu et al. (2019)
Lung (NSCLC)	miR-144/PRR11 axis	H1975 and A549	Δ DLX6-AS1: ↓ tumor cell proliferation, ↓migration, ↓invasion, and ↑apoptosis	Huang et al. (2019)
	miR27b3p/GSPT1 axis	CALU3, CALU6, A549, H1299, and HBE	Δ DLX6-AS1: ↓proliferation, ↓migration, and ↓invasion	Sun et al. (2019)
Ewing’s sarcoma	miR-124-3p/CDK4 axis	SK-ES-1, A673, RD-ES, and mesenchymal stem cells (MSCs)	Δ DLX6-AS1: ↓ tumor cell proliferation, and ↑apoptosis	Lei et al. (2019)

Δ: knockdown or silencing, ↓: decrease or repression, ↑: increase or stimulation, ↑↑: overexpression, CSCs: cancer stem cells.

## Impact of DLX6-AS1 in Enhancement of Tumor Growth

Experiments in animal models have confirmed oncogenic role of DLX6-AS1. It is expected that DLX6-AS1 overexpression or silencing increases or suppresses malignant features of cancer cells in xenograft models, respectively. To examine this claim, treated cells; either overexpressing or with silenced for DLX6-AS1; have been injected to the animals; mainly BALB/c nude mice, and then tumor size or volume, and metastasis in expected organ have been checked at certain intervals. Changes in chemosensitivity have also been assessed occasionally. Decreased tumor growth and metastasis, and also chemoresistance have been reported under DLX6-AS1 knockdown conditions in animal studies. Opposite findings have been reported when DLX6-AS1 was overexpressed in injected cancer cells to the nude mice. Taken together, these findings demonstrate oncogenic role of DLX6-AS1 in tumor progression and metastasis in animal studies are consistent with the results of cell studies (Table 2).

**TABLE 2:** Effects of DLX6-AS1 on tumor growth and metastasis in animal studies.

Cancer type	Animal models	Function	References
HCC	BALB/c nude mice	Δ DLX6-AS1: ↓ tumor growth	Liu et al. (2020a)
	BALB/c nude mice	Δ DLX6-AS1: ↓ tumor growth	Zhang et al. (2017)
Pancreas	BABL/c athymic nude mice	Δ DLX6-AS1: ↓ tumor growth	An et al. (2018)
	BABL/c athymic nude mice	Δ DLX6-AS1: ↓ tumor growth, and ↓metastasis	Yang et al. (2019a)
Prostate	BALB/c nude mice	Δ DLX6-AS1: ↓ tumor growth	Zhu et al. (2021)
	SCID mice	↑↑ DLX6-AS1: ↑tumor growth and ↑lymph node metastasis	Zhao et al. (2020b)
Neuroblastoma	BALB/c nude mice	Δ DLX6-AS1: ↓ tumor growth	Jia et al. (2020)
	BALB/c nude mice	Δ DLX6-AS1: ↓ tumor growth	Han et al. (2020)
	BALB/c nude mice	Δ DLX6-AS1: ↓ tumor growth	Li et al. (2020b)
	BALB/c nude mice	Δ DLX6-AS1: ↓ tumor growth	Li et al. (2019b)
Glioma	Male nude mic	Δ DLX6-AS1: ↓ tumor growth	Zhang et al. (2019b)
Endometria	32 healthy nude mice	Δ DLX6-AS1: ↓ tumor growth	Zhao and Xu, (2020)
Cervix	BALB/c nude mice	Δ DLX6-AS1: ↓ tumor growth	Xie et al. (2020)
Breast (TNBC)	BALB/c nude mice	Δ DLX6-AS1: ↓ tumor growth, and ↓chemoresistance to cisplatin	Du et al. (2020)
Ovaries	BALB/c nude mice	Δ DLX6-AS1: ↓ tumor growth	Kong and Zhang, (2020)
Bladder	BALB/c nude mice	Δ DLX6-AS1: ↓ tumor growth	Zhao et al. (2020a)
	Male nude mice	Δ DLX6-AS1: ↓ tumor growth	Guo et al. (2019)
Larynx	BALB/c nude mice	Δ DLX6-AS1: ↓ tumor growth	Liu et al. (2020b)
Stomach	BALB/c nude mice	Δ DLX6-AS1: ↓ tumor growth	Qian et al. (2021)
Osteosarcoma	BALB/c nude mice	Δ DLX6-AS1: ↓ tumor growth	Zhang et al. (2018)
	BALB/c nude mice	Δ DLX6-AS1: ↓ tumor growth	Zhang et al. (2019b)
Lung (NSCLC)	BALB/c nude mice	Δ DLX6-AS1: ↓ tumor growth	Huang et al. (2019)
	BALB/c nude mice	Δ DLX6-AS1: ↓ tumor growth	Sun et al. (2019)
Colorectal	Female nude mice	Δ DLX6-AS1: ↓ tumor growth	Zhang et al. (2019a)
Liver	NOD-SCID mice	Δ DLX6-AS1: ↓tumorigenesis and ↓tumor growth	Wu et al. (2019)
Kidney (RCC)	BALB/c nude mice	Δ DLX6-AS1: ↓ tumor growth	Zeng et al. (2017)

## Impact of DLX6-AS1 on Survival of Patients With Different Types of Cancers

Cancerous tissues resected from patients have shown significantly increased expression of DLX6-AS1 compared to matched normal adjacent tissues (NATs) and healthy people in microarray analysis and qRT-PCR. In non-small cell lung cancer (NSCLC), DLX6-AS1 high expression levels were found to be positively associated with advanced clinicopathological features including higher disease stage, tumor metastasis to lymph nodes and also weak differentiation of cancer cells in patients (Zhang et al., 2019c). Also, Guo et al. (2019) demonstrated high DLX6-AS1 expression in bladder cancer patients with advanced TNM stage, positive lymph node and distant metastases. Survival analysis *via* Kaplan-Meier curve has shown association between high DLX6-AS1 expression and shorter overall survival (OS), and/or disease-free survival (DFS) in several types of cancer like HCC (Liu et al., 2020a; Zhang et al., 2017), gastric cancer (Qian et al., 2021; Fu et al., 2019), glioma (Zhang et al., 2019b), breast cancer (Zhao et al., 2019), and several others (Table 3). Competitive endogenous RNA (ceRNA) network analysis has demonstrated reliability of DLX6-AS1 along with three other lncRNAs and two more miRNAs in a signature as prognostic biomarkers in HCC patients (Long et al., 2019). Ding et al. (2021) showed serum exosomal levels of DLX6-AS1 can act as a prognostic biomarker in cervical cancer patients. Also, multivariate cox regression has shown that DLX6-AS1 is an independent prognostic factor for survival in a number of cancers such as gastric cancer (Qian et al., 2021), osteosarcoma (Zhang et al., 2018), and ovarian cancer (Zhao and Liu, 2019). Furthermore, a value of 0.795 for area under curve (AUC) in receiver operating characteristic (ROC) curve has shown acceptable efficiency of DLX6-AS1 in diagnosis of glioma (Zhang et al., 2019b). Taken together, according to the clinical data, DLX6-AS1 is suggested as a potential prognostic biomarker for different types of human cancer and a putative factor to manage cancerous patients.

**TABLE 3:** Clinical prognostic importance of DLX6-AS1 in human cancers.

Cancer type	Clinical samples	Expression change in tumor tissues compared to normal tissues	Kaplan-Meier analysis	Multivariate cox regression	References
HCC	85 cancerous patients and matched NATs	Up	Patients with high DLX6-AS1 expression had poor OS compared to those with lower levels	--	Liu et al. (2020a)
	60 cancerous patients and matched NATs	Up	High DLX6-AS1 expression levels were correlated with poor OS in HCC patients compared to low levels	--	Zhang et al. (2017)
Larynx	43 cancerous patients and matched NATs	Up	Patients with high DLX6-AS1 expression had shorter OS compared to those with lower levels	--	Liu et al. (2020b)
Stomach	60 cancerous tissues and 28 NATs	Up	High DLX6-AS1 expression levels were associated with poor OS.	DLX6-AS1 expression is an independent predictor of poor prognosis	Qian et al. (2021)
	375 cancerous tissues and 32 NATs	Up	--	--	Liang et al. (2020)
	62 cancerous tissues and matched NATs	Up	High DLX6-AS1 expression levels correlated with shorter survival in gastric cancer patients compared to those with low levels	--	Fu et al. (2019)
Glioma	36 cancerous tissues and matched NATs	Up	Patients with high DLX6-AS1 expression levels exhibited shorter OS compared to those with low levels	--	Zhang et al. (2019b)
Osteosarcoma	80 cancerous tissues and matched NATs	Up	High DLX6-AS1 expression levels were correlated with shorter OS in osteosarcoma patients compared to low levels	DLX6-AS1 expression level is an independent prognostic factor	Zhang et al. (2018)
Breast	45 cancerous tissues and matched NATs	Up	High DLX6-AS1 expression levels were correlated with shorter OS in osteosarcoma patients compared to low levels	--	Zhao et al. (2019)
Pancreas	60 cancer tissues and matched NATs	Up	Patients with low DLX6-AS1 expression levels exhibited higher survival rate compared to those with high levels	--	Yang et al. (2019a)
	84 cancer tissues and matched NATs	Up	--	--	An et al. (2018)
Prostate	20 cancer tissues and matched NATs	Up	--	--	Zhu et al. (2021)
	32 cancerous patients and 28 patients with benign prostate hyperplasia	Up	--	--	Zhao et al. (2020b)
Neuroblastoma	20 cancer tissues and matched NATs	Up	--	--	Jia et al. (2020)
	31 cancer tissues and matched NATs	Up	--	--	Han et al. (2020)
	70 cancer tissues and matched NATs	Up	High DLX6-AS1 expression levels were significantly associated with shorter OS in neuroblastoma patients compared to those with low levels	--	Li et al. (2020b)
	88 cancer tissues and matched NATs	Up	High DLX6-AS1 expression levels were correlated with shorter OS in neuroblastoma patients compared to those with low levels		Li et al. (2019b)
Endometria	78 cancer tissues and matched NATs	Up	--	--	Zhao and Xu, (2020)
Breast (TNBC)	47 cancerous tissues and matched NATs	Up	--	--	Du et al. (2020)
Ovaries	50 cancerous tissues and matched NATs	Up	--	--	Kong and Zhang, (2020)
	128 cancerous tissues and matched NATs	Up	Patients with high DLX6-AS1 expression levels had shorter OS and DFS compared to those with low levels	DLX6-AS1 expression is an independent prognostic factor for survival in ovarian cancer patients	Zhao and Liu, (2019)
Bladder	60 cancerous tissues and matched NATs	Up	--	--	Zhao et al. (2020a)
	54 cancerous tissues and matched NATs	Up	--	--	Guo et al. (2019)
Colorectal	76 cancerous tissues and matched NATs	Up	--	--	Kong et al. (2020)
	60 cancerous tissues and matched NATs	Up	--	--	Zhang et al. (2019a)
Larynx (LSCC)	23 cancerous tissues and matched NATs	Up	--	--	Yang et al. (2019b)
Osteosarcoma	40 cancerous tissues and matched NATs	Up	--	--	Zhang et al. (2019b)
Lung (NSCLC)	48 cancerous tissues and matched NATs	Up	--	--	Huang et al. (2019)
	51 cancerous tissues and matched NATs	Up	--	--	Sun et al. (2019)
Nasopharynx	72 cancerous tissues and matched NATs	Up	--	--	Yang et al. (2020)
Esophagus	73 cancerous tissues and matched NATs	Up	--	--	Zhang et al. (2019c)
Liver	30 cancerous tissues and matched NATs	Up	--	--	Li et al. (2019a)
Cervix	78 cancerous tissues and matched NATs	Up	--	--	Long et al. (2019)
Kidney (RCC)	15 cancerous tissues and matched NATs	Up	--	--	Zeng et al. (2017)
Ewing’s sarcoma	20 cancerous tissues and matched NATs	Up	--	--	Lei et al. (2019)

## Discussion

LncRNAs are a heterogeneous group of ncRNAs with characteristic size of more than 200 nucleotides. An increasing number of lncRNAs have been found to be dysregulated in many human diseases particularly cancer. However, their role in carcinogenesis is not precisely understood. DLX6-AS1 is an lncRNAs which has been unveiled to be up-regulated in a various number of cancers. In different cell studies, DLX6-AS1 has shown oncogenic role *via* promoting oncogenic phenotype of cancer cell lines. Increase in tumor cell proliferation, migration, invasion, and EMT while suppressing apoptosis in cancer cells are the effects of DLX6-AS1 in the development and progression of cancer. Silencing experiments using specific shRNA against DLX6-AS1 have shown suppression of tumorigenic potential. Similar pattern of expression in different types of cancer originated from various tissues not only reveals its universal function in the tumorigenesis, but also emphasizes the suitability of therapeutic modalities against this lncRNA for a wide range of human malignancies.

In the majority of cell experiments, mediator miRNAs have been identified which are negatively regulated by DLX6-AS1, and they regulate expression of a number of transcription factors, eventually affecting signaling pathways involved in carcinogenesis. These pathways form axes through which DLX6-AS1 regulates transcription factors, and/or signaling pathways eventually promotes carcinogenicity of cancer cells. Identification of functional routes of DLX6-AS1 effects in the carcinogenesis is an important step toward design of targeted therapies in cancer. It is also important to mention that these therapies should not affect pathways with crucial roles in the physiological features of normal cells.

Xenograft animal studies also have confirmed enhancing effect of DLX6-AS1 on tumor growth and metastasis. Clinical evaluations in cancerous patients have shown increased expression of DLX6-AS1 in tumor tissues compared to healthy tissues. High DLX6-AS1 expression has shown positive association with advanced clinicopathological features in cancerous patients. Survival analyses have demonstrated correlation between high DLX6-AS1 expression and shorter survival. In cox regression analysis, DLX6-AS1 has been suggested as an independent prognostic factor for patients with various types of cancer.

Animal and cell line studies have confirmed that therapeutic modalities targeting DLX6-AS1 can effectively reduce tumorigenic potential of malignant cells, induce their apoptosis and diminish tumor size and burden. However, the efficacy and safety of these methods have not been evaluated in the clinical settings.

Taken together, these findings demonstrate carcinogenic role of DLX6-AS1 in the development and progression of different human cancers suggesting diagnostic and prognostic potentials of DLX6-AS1 in human cancers. Known role of up-regulated DLX6-AS1 in cancer tissues and clinical samples also suggest therapeutic potentials in finding treatments for different types of cancer *via* targeting DLX6-AS1. Further studies are required to utilize diagnostic, prognostic, and therapeutic potentials of DLX6-AS1 in clinical settings. Moreover, measurement of DLX6-AS1 levels in biofluids is an important step towards identification of non-invasive routes for diagnostic purposes.
